# Supplementary material for: Clinical evolution and medical resource utilization in adult patients with respiratory syncytial virus infection at a community hospital in Argentina
Source: PLoS One. 2025 May 22;20(5):e0324735. doi: 10.1371/journal.pone.0324735 (PMC12097560; doi:10.1371/journal.pone.0324735)
Supplement: S1 File — (DOCX) [file pone.0324735.s001.docx]

**Supporting information**

| **Table S1.** Annual Breakdown of RSV Testing, Positivity, and Hospitalizations   \| **Year** \| **RSV Positivity (Positive Cases / Total Tests)** \| **Hospitalization Rate (Hospitalizations / RSV Positive Cases)** \| \| --- \| --- \| --- \| \| 2010 \| 0.00% (0 / 3) \| 0.00% (0 / 0) \| \| 2011 \| 25.00% (1 / 4) \| 100.00% (1 / 1) \| \| 2012 \| 0.00% (0 / 14) \| 0.00% (0 / 0) \| \| 2013 \| 4.35% (1 / 23) \| 100.00% (1 / 1) \| \| 2014 \| 9.68% (6 / 62) \| 66.67% (4 / 6) \| \| 2015 \| 1.95% (3 / 154) \| 66.67% (2 / 3) \| \| 2016 \| 1.72% (4 / 232) \| 75.00% (3 / 4) \| \| 2017 \| 1.98% (5 / 252) \| 60.00% (3 / 5) \| \| 2018 \| 1.66% (4 / 241) \| 25.00% (1 / 4) \| \| 2019 \| 2.57% (12 / 467) \| 50.00% (6 / 12) \| \| 2020 \| 0.69% (2 / 290) \| 100.00% (2 / 2) \| \| 2021 \| 6.54% (10 / 153) \| 90.00% (9 / 10) \| \| 2022 \| 3.14% (13 / 414) \| 84.62% (11 / 13) \| \| 2023 \| 5.61% (37 / 659) \| 78.38% (29 / 37) \| \| Total \| 3.30% (98 / 2968) \| 73.47% (72 / 98) \|   RSV, respiratory syncytial virus  **Table S2.** Resource utilization in patients hospitalized with RSV and Influenza | | |
| --- | --- | --- | --- | --- | --- | --- | --- | --- | --- | --- | --- | --- | --- | --- | --- | --- | --- | --- | --- | --- | --- | --- | --- | --- | --- | --- | --- | --- | --- | --- | --- | --- | --- | --- | --- | --- | --- | --- | --- | --- | --- | --- | --- | --- | --- | --- | --- | --- | --- | --- |
|  | **RSV=72 n (%)** | **Influenza =226 n (%)** |
| Antibiotics | 54 (75) | 173 (76.55) |
| Corticosteroids | 49 (68.05) | 144 (63.72) |
| Oxygen therapy | 32 (44.44) | 95 (42.04) |
| Chest X ray | 46 (63.88) | 118 (52.21) |
| Computerized tomography scan | 27 (37.50) | 70 (30.97) |
| ICU admission | 27 (37.50) | 64 (28.32) |
| NIV | 19 (26.38) | 50 (22.12) |
| MV | 8 (11.11) | 31 (13.72) |
| Bronchoalveolar lavage | 5 (6.94) | 22 (9.74) |

RSV, respiratory syncytial virus; ICU, intensive care unit; NIV, non-invasive ventilation; MV, mechanical ventilation

| **Table S3.** Resource utilization in patients hospitalized with RSV and Influenza | | |
| --- | --- | --- |
| **Mean (SD)** | **RSV (n=72)** | **Influenza (n=226)** |
| Duration of hospital stay, days | 12.18 (12.91) | 10.85 (19.10) |
| Oxygen requirement, days | 4.17 (6.67) | 3.56 (7.13) |
| Antibiotic, days | 7.99 (9.72) | 8.5 (17.78) |
| Corticosteroids, days | 9.06 (10.69) | 7.89 (18.65) |
| ICU, days | 5.49 (11.73) | 4.05 (12.36) |
| NIV, days | 2.96 (6.39) | 1.66 (4.33) |
| MV, days | 2.43 (9.52) | 2.28 (10.14) |

RSV, respiratory syncytial virus; ICU, intensive care unit; NIV, non-invasive ventilation; MV, mechanical ventilation

**Access link to the Dataset:**

https://docs.google.com/spreadsheets/d/1rxAyVUOXAeXDxyWZORB83OZI0moMA1vIx7oI4bNSh0w/edit?usp=sharing
