# Supplementary material for: Clinical evolution and medical resource utilization in adult patients with respiratory syncytial virus infection at a community hospital in Argentina
Source: PLoS One. 2025 May 22;20(5):e0324735. doi: 10.1371/journal.pone.0324735 (PMC12097560; doi:10.1371/journal.pone.0324735)
Supplement: Attachment — Submitted filename: Response to Reviewers letter PONE-D-24-46783.docx [file pone.0324735.s002.docx]

Dear Editors and Reviewers,

We appreciate the chance to address an important clarification regarding our submission. We apologize for the error in our previous submission, where we inadvertently uploaded the ethics approval document from a different study.

To clarify, we have the official ethics approval document for this study from the Buenos Aires province, granted through the Plataforma de Registro Informatizado de Investigaciones en Salud (PRIISA.BA) in January 2024, in full compliance with Argentine protocol registration standards, as stated in the manuscript. We also have the original email correspondence containing the approval and documentation from PRIISA.BA.

In this new submission, we have attached the correct ethics approval document, and we respectfully request a re-evaluation of the manuscript with this information accurately represented.

Thank you for your understanding and consideration.

Sincerely,

Dr. Agustín Bengolea

Agustin M. Bengolea, MD.

Corresponding Author

Hospital Aleman - Av. Pueyrredon 1640. Ciudad de Buenos Aires. Argentina. CP 1118

Email: agustinmbengolea@gmail.com
