# Supplementary material for: Clinical evolution and medical resource utilization in adult patients with respiratory syncytial virus infection at a community hospital in Argentina
Source: PLoS One. 2025 May 22;20(5):e0324735. doi: 10.1371/journal.pone.0324735 (PMC12097560; doi:10.1371/journal.pone.0324735)
Supplement: Attachment — Submitted filename: Response to reviewers.docx [file pone.0324735.s003.docx]

Dear Editor,

We thank you for the valuable comments and constructive feedback that have significantly improved this manuscript. Below, we address each comment in detail.

**Journal Requirements:**

1. *1. Please ensure that your manuscript meets PLOS ONE's style requirements, including those for file naming.*

**Addressed**

2. *Thank you for stating the following financial disclosure: “The research described herein was supported by Pfizer Inc. LRA, CGV, and NZ are employed by Pfizer Argentina SRL. AB is an employee of Hospital Alemán, which received financial support from Pfizer Inc. for this study (including manuscript preparation).” Please state what role the funders took in the study. If the funders had no role, please state: "The funders had no role in study design, data collection and analysis, decision to publish, or preparation of the manuscript." If this statement is not correct you must amend it as needed. Please include this amended Role of Funder statement in your cover letter; we will change the online submission form on your behalf.*

**Included in the cover letter**

3. *We noted in your submission details that a portion of your manuscript may have been presented or published elsewhere. [A poster presenting preliminary results from this study is scheduled for presentation at the upcoming ISPOR Europe conference in Barcelona. The data and analyses in this manuscript encompass a comprehensive, final dataset with additional insights and detailed analyses that go beyond the scope of the poster. Accordingly, this submission does not constitute dual publication, as it provides an expanded and complete version of the findings.] Please clarify whether this [conference proceeding or publication] was peer-reviewed and formally published. If this work was previously peer-reviewed and published, in the cover letter please provide the reason that this work does not constitute dual publication and should be included in the current manuscript.*

**Included in the cover letter**

4. *In this instance it seems there may be acceptable restrictions in place that prevent the public sharing of your minimal data. However, in line with our goal of ensuring long-term data availability to all interested researchers, PLOS’ Data Policy states that authors cannot be the sole named individuals responsible for ensuring data access (*[*http://journals.plos.org/plosone/s/data-availability#loc-acceptable-data-sharing-methods*](http://journals.plos.org/plosone/s/data-availability#loc-acceptable-data-sharing-methods)*). Data requests to a non-author institutional point of contact, such as a data access or ethics committee, helps guarantee long term stability and availability of data. Providing interested researchers with a durable point of contact ensures data will be accessible even if an author changes email addresses, institutions, or becomes unavailable to answer requests. Before we proceed with your manuscript, please also provide non-author contact information (phone/email/hyperlink) for a data access committee, ethics committee, or other institutional body to which data requests may be sent. If no institutional body is available to respond to requests for your minimal data, please consider if there any institutional representatives who did not collaborate in the study, and are not listed as authors on the manuscript, who would be able to hold the data and respond to external requests for data access? If so, please provide their contact information (i.e., email address). Please also provide details on how you will ensure persistent or long-term data storage and availability.*

**Included in the cover letter**

5. *Please include captions for your Supporting Information files at the end of your manuscript, and update any in-text citations to match accordingly. Please see our Supporting Information guidelines for more information:* [*http://journals.plos.org/plosone/s/supporting-information*](http://track.editorialmanager.com/CL0/http:%2F%2Fjournals.plos.org%2Fplosone%2Fs%2Fsupporting-information/1/010f019448cab949-23fe77f0-3f70-4aa7-8f3d-7689465c5215-000000/kFpdbbPYazIb8jD2ERsZTgj_FnqIJrfsNdGoUxYToSY=193)*.*

**Included in the manuscript**

6. *Please review your reference list to ensure that it is complete and correct. If you have cited papers that have been retracted, please include the rationale for doing so in the manuscript text, or remove these references and replace them with relevant current references. Any changes to the reference list should be mentioned in the rebuttal letter that accompanies your revised manuscript. If you need to cite a retracted article, indicate the article’s retracted status in the References list and also include a citation and full reference for the retraction notice.*

**All references were reviewed and no cited papers have been retracted**

7. *In the online submission form, you indicated that "All data generated or analyzed during this study are included in this published article/as supplementary information files. The datasets generated during and/or analyzed during the current study are available from the corresponding author on reasonable request."

   All PLOS journals now require all data underlying the findings described in their manuscript to be freely available to other researchers, either
   1. In a public repository,
   2. Within the manuscript itself, or
   3. Uploaded as supplementary information.

   This policy applies to all data except where public deposition would breach compliance with the protocol approved by your research ethics board. If your data cannot be made publicly available for ethical or legal reasons (e.g., public availability would compromise patient privacy), please explain your reasons on resubmission and your exemption request will be escalated for approval.*

**Included the link to the public repository in the supplementary information**

8. *We note that several of your files are duplicated on your submission. Please remove any unnecessary or old files from your revision, and make sure that only those relevant to the current version of the manuscript are included.*

**Addressed**

**Reviewers' comments:
*Reviewer #1:*** *This article presents data on the RSV burden in adults from a single center in Argentina. Although it adds little to the existing body of evidence on the topic, it is well-written. A few minor comments.*

1. *Table 1: add the % of patients with at least one chronic condition
   For influenza patients: Add the % of CTC and ATB use. Add if available % of flu vaccination.*

**The percentages of corticosteroid and antibiotic use for influenza and RSV are available in the supplementary information. Unfortunately, we do not have data on flu vaccination rates, as this information was not recorded in the medical histories.**

2. *Would it be possible to add information on co-infections for the RSV patients?*

**Yes, we have included them in the results section. The relevant text states: “Among the 72 hospitalized RSV patients, 45 (62.5%) received conventional inpatient care, while 27 (37.5%) required ICU admission.** **Additionally, 26 patients (36.1%) had a bacterial co-infection: 6 (23.1%) from blood, 12 (46.2%) from urine, 7 (26.9%) from sputum or respiratory cultures, and 1 (3.8%) from a stool sample.”**

3. *I am surprised by the high rate of Antibiotic prescriptions in these patients. (Is this due to bacterial co-infection? Or misuse ?) Can you comment on this rate and the high CTC rate as well?*

**Included in the discussion section: “The high rate of antibiotic use observed in our cohort, with 75% of patients receiving antibiotics, is noteworthy. While this may partly reflect the severity of illness in patients hospitalized with RSV and the potential for bacterial co-infection, it also raises concerns regarding potential overuse. Such extensive prescribing warrants scrutiny, particularly in the context of RSV, a predominantly viral pathogen where routine antibiotic use may lack clinical justification.”**

***Reviewer #2:***

1. *The authors describe differences between the two cohorts (syncytial virus and influenza) and conclude with some differences between them. ¿Are those differences significant? ¿It would be appropriate to explain why a statistical study was not carried out between the two cohorts?.*

**No statistical analysis was conducted to compare the RSV and influenza cohorts, as this was an exploratory analysis. This has been clarified in the methods section: “No statistical analysis was performed to compare the RSV and influenza cohorts, as this was an exploratory analysis. The purpose of this comparison was to provide descriptive insights into differences in medical resource utilization and patient outcomes between these two groups.** **A statistical comparison would be misleading”**

2. *The authors report that 27 patients required ICUs; nineteen received NIV and 8 received mechanical ventilation. Then, they say that 31 patients met the criterion of "severe disease", which includes the compound of non-invasive ventilation and admission to the ICU. Why that difference of 4 patients?*

**The difference arises because not all patients who received non-invasive ventilation (NIV) were in the ICU. At the Hospital Alemán, NIV can be provided outside the ICU setting, which accounts for the discrepancy in the numbers.**

***Reviewer #3****:*

*This is an important study on RSV in Argentina, for which there is little published evidence, especially regarding the burden of disease among adults and the healthcare utilization.
The authors conducted a retrospective cohort study for which they identified all RSV positive cases in a 14 year period in a single Argentinian center. They also report the number of all patients that were subjected to viral testing. The manuscript is well structured and written and with an easy to follow, logical chain of thoughts. I will give comments in chronological order and will highlight aspects that I deem critical that should be revised to improve the quality of the work. Of note, the study was financially supported by Pfizer, and three of the authors are employed by the company.*

1. *Title: no comments*
2. *Abstract: I think the finding that 75% were treated with antibiotics is absolutely worrying. This should be picked up in the conclusions section as well, depending on further investigations/analyses that I comment on in the results part.*

**We have included in the manuscript and the abstract: “Antibiotics (75%) and corticosteroids (68.05%) were commonly used, likely reflecting the severity of clinical presentation or the potential for bacterial coinfection.” “The high rate of antibiotic use observed in our cohort, with 75% of patients receiving antibiotics, is noteworthy. While this may partly reflect the severity of illness in patients hospitalized with RSV and the potential for bacterial co-infection, it also raises concerns regarding potential overuse. Such extensive prescribing warrants scrutiny, particularly in the context of RSV, a predominantly viral pathogen where routine antibiotic use may lack clinical justification.”**

3. *Introduction: no comments*
4. *Methods: please briefly explain what the algorithm is for microbiological/viral testing, as the overall number that you report in the results for such a long period of time is rather low (212 per year?)*

**No algorithm for microbiological/viral testing is applied at the Hospital Alemán. Testing is typically reserved for patients with severe respiratory symptoms or those requiring hospitalization. This has been clarified in the methods section: “****Testing is usually reserved for patients presenting with severe respiratory symptoms or those requiring hospitalization.”**

5. *Results: please provide numbers also per year, so potential time trends, both with regard to total tests ordered and positive tests, can be assessed*

**Included in the results and in the supplementary information section: “The annual breakdown of testing, RSV-positive cases, and hospitalizations has been moved to a table in the appendix for clarity and conciseness. Please refer to Table S1 in the for detailed year-by-year data.**

**Table S1.**

6. *Results: I think one crucial information that is lacking at the moment is information on the reasoning of antibiotic treatment. It could be helpful to include data on microbiological diagnostics in these cases (they are not that many, so it should be feasible), to approximate whether there is a reasonable number of patients with a bacterial co-infection. I presume that anyhow the vast majority will be unneccesary overtreatment of viral cases.*

**Included in the results section: “Among the 72 hospitalized RSV patients, 45 (62.50%) received conventional inpatient care, while 27 (37.50%) required ICU admission. Additionally, 27 patients (37.5%) had a bacterial co-infection.”**

**Table S1.** Annual Breakdown of RSV Testing, Positivity, and Hospitalizations

| **Year** | **RSV Positivity (Positive Cases / Total Tests)** | **Hospitalization Rate (Hospitalizations / RSV Positive Cases)** |
| --- | --- | --- |
| 2010 | 0.00% (0 / 3) | 0.00% (0 / 0) |
| 2011 | 25.00% (1 / 4) | 100.00% (1 / 1) |
| 2012 | 0.00% (0 / 14) | 0.00% (0 / 0) |
| 2013 | 4.35% (1 / 23) | 100.00% (1 / 1) |
| 2014 | 9.68% (6 / 62) | 66.67% (4 / 6) |
| 2015 | 1.95% (3 / 154) | 66.67% (2 / 3) |
| 2016 | 1.72% (4 / 232) | 75.00% (3 / 4) |
| 2017 | 1.98% (5 / 252) | 60.00% (3 / 5) |
| 2018 | 1.66% (4 / 241) | 25.00% (1 / 4) |
| 2019 | 2.57% (12 / 467) | 50.00% (6 / 12) |
| 2020 | 0.69% (2 / 290) | 100.00% (2 / 2) |
| 2021 | 6.54% (10 / 153) | 90.00% (9 / 10) |
| 2022 | 3.14% (13 / 414) | 84.62% (11 / 13) |
| 2023 | 5.61% (37 / 659) | 78.38% (29 / 37) |
| Total | 3.30% (98 / 2968) | 73.47% (72 / 98) |

RSV, respiratory syncytial virus

7. *Discussion: The issue of overtreatment could be picked up even more here*

**Included in the discussion section: “The high rate of antibiotic use observed in our cohort, with 75% of patients receiving antibiotics, is noteworthy. While this may partly reflect the severity of illness in patients hospitalized with RSV and the potential for bacterial co-infection, it also raises concerns regarding potential overuse. Such extensive prescribing warrants scrutiny, particularly in the context of RSV, a predominantly viral pathogen where routine antibiotic use may lack clinical justification.**

**Bacterial co-infection was confirmed through respiratory cultures in 26.9% of patients, a prevalence consistent with findings from a systematic review[14] of bacterial co-infections in influenza, which reported a pooled prevalence of 22.5% (95% CI: 16.5–30.0). While these results highlight the role of bacterial co-infections in severe respiratory viral illnesses, they fall short of explaining the high rates of antibiotic use observed in our cohort. This discrepancy suggests that antibiotics may have been prescribed inappropriately, in line with findings from the systematic review[15], which reported that 36.9% of antibiotic prescriptions for respiratory infections in emergency department settings were unnecessary.**

**These findings emphasize the importance of targeted interventions to address the misuse of antibiotics in the management of respiratory viral infections. Strengthening diagnostic capabilities, particularly with rapid tests for bacterial pathogens, is essential. Moreover, implementing evidence-based guidelines, coupled with education for clinicians on judicious antibiotic prescribing, can help mitigate inappropriate use. Future research should explore the behavioral and systemic factors driving these prescribing patterns and evaluate the impact of stewardship programs in reducing misuse. By addressing these gaps, we can optimize the management of respiratory infections while preserving the efficacy of antibiotics for patients with confirmed bacterial infections..”**
