# Supplementary material for: Clinical evolution and medical resource utilization in adult patients with respiratory syncytial virus infection at a community hospital in Argentina
Source: PLoS One. 2025 May 22;20(5):e0324735. doi: 10.1371/journal.pone.0324735 (PMC12097560; doi:10.1371/journal.pone.0324735)
Supplement: Attachment — Submitted filename: Response_to_Reviewers_auresp_2.docx [file pone.0324735.s004.docx]

Dear Reviewers,

We sincerely thank you for your valuable comments and constructive feedback, which have contributed to significantly improving the quality of our manuscript. Below, we provide detailed responses to each of your points.

Reviewer #2:

1. *In Table2, the number of patients recquired NIV were 19, but in your response 4 received NIV out of the ICU. Thus ,the number of NIV in Table 2 must be 23 , with adjusted mean days (SD)*

Response to reviewers:

**Table 2 summarizes the results for the entire study population, including both patients who required ICU admission and those who did not. As previously mentioned, at Hospital Alemán, non-invasive ventilation (NIV) can be administered not only in the ICU but also in general wards.**

**As stated in the manuscript: “Nineteen patients (19/72; 26.38%) required NIV, with a mean duration of 2.96 days (SD = 6.39); eight patients (8/72; 11.11%) required mechanical ventilation (MV), with a mean duration of 2.43 days (SD = 9.52). Among the 27 patients (37.50%) admitted to the ICU, the mean ICU stay was 5.49 days (SD = 11.73). Four patients (5.55%) with RSV-positive tests died during hospitalization. Severe disease, defined as the composite of NIV and ICU admission, was observed in 31 patients (43.10%).”**

**In our previous response, we stated: “The difference arises because not all patients who received non-invasive ventilation (NIV) were in the ICU. At Hospital Alemán, NIV can be provided outside the ICU setting, which accounts for the discrepancy in the numbers.”**

**We now realize that our explanation may have led to some misunderstanding, and we thank the reviewer for pointing this out. To clarify, we have added the following footnote for the NIV to Table 2: *“Includes NIV at ICU and general hospital ward”***

Reviewer #3:

1. *One remaining issue is the antibiotic overtreatment. I think it still lacks contextualization. Is the rate observed in your cohort high, low or average? can you cite some studies for a better comparison?*

**We have included the following in the discussion section: “A recent Norwegian cohort study of 951 hospitalizations for confirmed viral respiratory tract infections, including RSV and influenza, found that 76% of patients received antibiotics despite no evidence of bacterial infection, and discontinuation of antibiotics within one day after viral diagnosis occurred in only 15.8% of cases[14]**

**These findings are strikingly similar to our own and reinforce the notion that empirical antibiotic use remains common, even in high-income settings with low background levels of antibiotic resistance.”**

2. *Furthermore, what do you mean with "a predominantly viral pathogen"??? RSV is a virus, period.*

**Thank you for pointing this out. You are correct—referring to RSV as a "predominantly viral pathogen" was inaccurate. We have corrected the sentence to: “Such extensive prescribing warrants scrutiny, particularly in the context of RSV, a viral pathogen for which routine antibiotic use may lack clinical justification.”**
